# Supplementary material for: Organization of DNA Replication Origin Firing in Xenopus Egg Extracts: The Role of Intra-S Checkpoint
Source: Genes (Basel). 2021 Aug 9;12(8):1224. doi: 10.3390/genes12081224 (PMC8394201; doi:10.3390/genes12081224)
Supplement: Supplementary file 1 [file genes-12-01224-s001.zip › S2.pdf]

Supplementary Material S2

The real DNA replication process is far more complex than any of the considered models. To explore how accurately MM5 can map a more complex process, we built, based on replication process in other eukaryotes [1–7] and our previous model [8], a more elaborate model (MM6) to generate *in silico* data with 8%, 19% and 53% global replicated fractions.

1 The MM6 model used to generate the *in silico* data

In MM6, localized potential origins were distributed with a uniform density $\rho = 1\ kb^{-1}$ and N_{dom} domains of size l_{dom} were randomly positioned along a genome of length $L = 10^5\ kb$. As in previous works, we assumed that at the start to S phase N_0 limiting factors were available for origin firing and their number, $N(t)$, increased during the course of S phase as $N(t) = N_0 + Jt$, and that each factor was sequestrated by new forks upon origin activation and released and made available again for origin firing upon coalescence of converging forks. Forks progressed at a constant velocity $v = 0.5\ kb.min^{-1}$. The probability of origin firing by encounter with a limiting factor was higher inside the domains $(P_0 + P_{dom})$ than outside them (P_0). In addition, origins outside but not inside the domains had a non-null probability P_{inhib} of being inhibited. Two local effects were allowed to act within a distance d_{fork} from active forks: P_0 was enhanced by P_{fork} and origin inhibition was relieved with a probability $P_{deinhib}$. We simulated 300 complete S phases using the 10 parameter values listed in Table S1, and extracted snapshots at 8%, 19% and 53% global replicated fractions. Each snapshot was considered as an independent sample and for each of them: i) the genome was randomly cut following the molecule length distribution presented in Figure 1 of materials and methods, ii) the data were reshaped as described in material and methods to account for the finite experimental resolution and iii) the distributions of $I(f)$, replicated fraction of single fibres, global fork density, eye-to-eye distances, gap lengths and eye lengths were determined.

Table S1. Values of MM5's parameters. These values are chosen arbitrarily.

Parameter	Value
N_0	107
$J(s^{-1})$	29
P_0	0.11
P_{inhib}	0.96
P_{fork}	0.28
$d(kb)$	94.91
N_{dom}	196
l_{dom}	192.39
$P_{deinhib}$	0.06
P_{dom}	0.73

2 Fitting the *in silico* data by MM5 model

By independently fitting the simulated profiles of each global replicated fraction, we implicitly assume that samples could originate from separated experiments, hence MM5 parameters values are possibly different for each global replicated fraction. This allows us to accurately reproduce observations from each sample (Figures S1, S2 and S3).

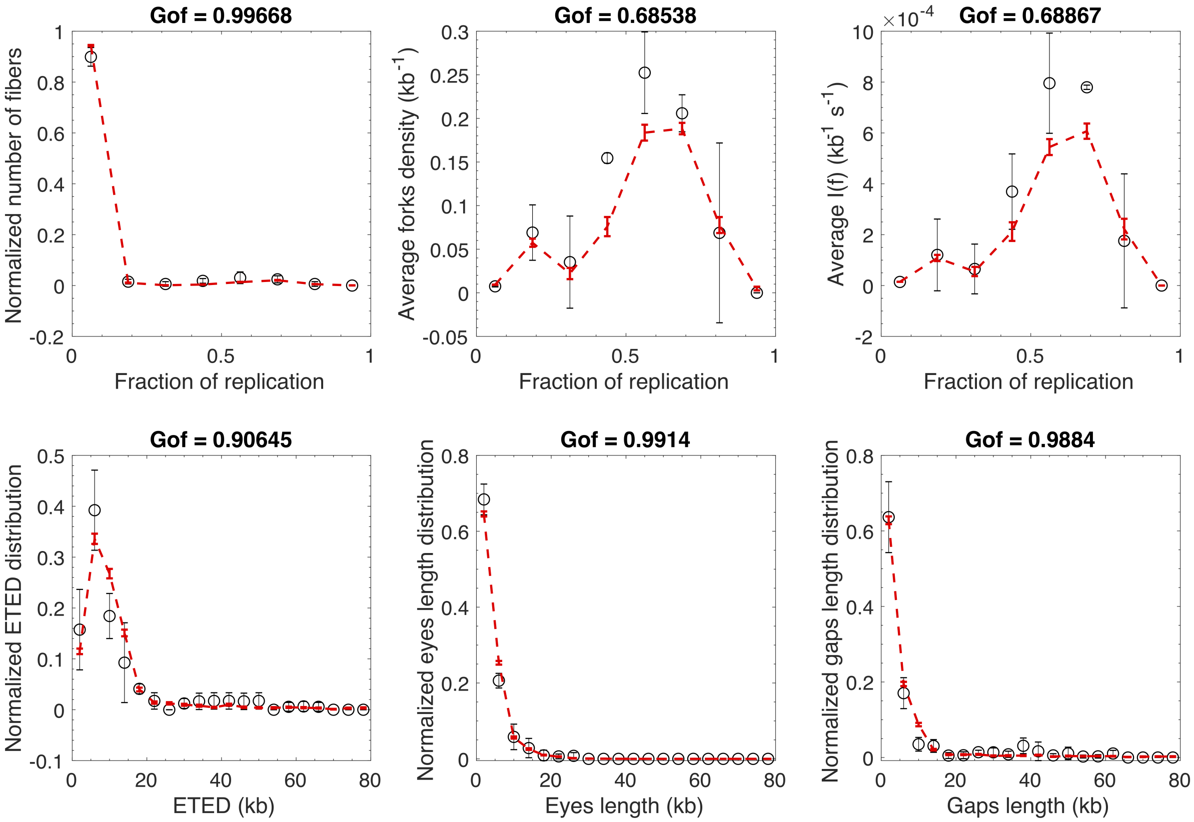

Figure S1. Modeling 8% global replicated fraction simulated data with discrete MM5 model. Open circles are simulated data and the red dashed line is the fit. $GoF_{global} = 0.96$

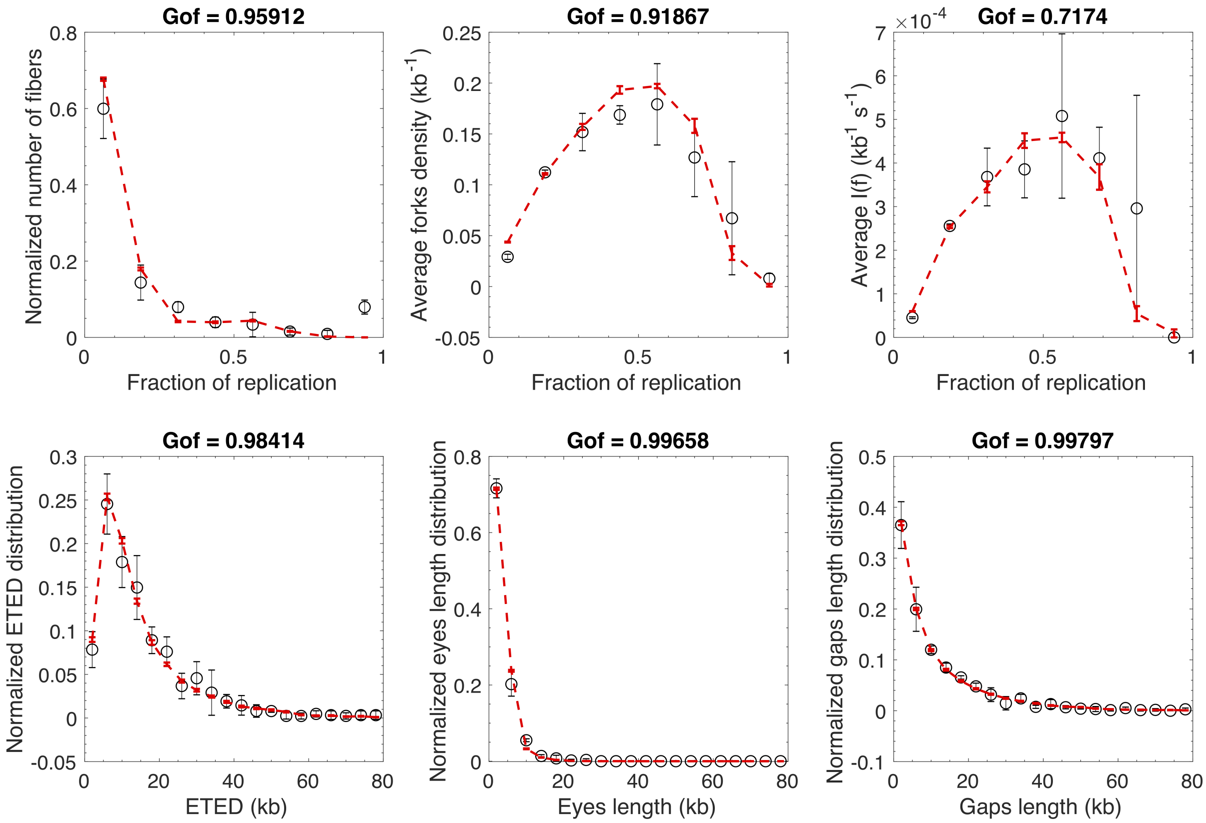

Figure S2. Modeling 19% global replicated fraction simulated data with discrete MM5 model. Open circles are simulated data and the red dashed line is the fit. $GoF_{global} = 0.97$

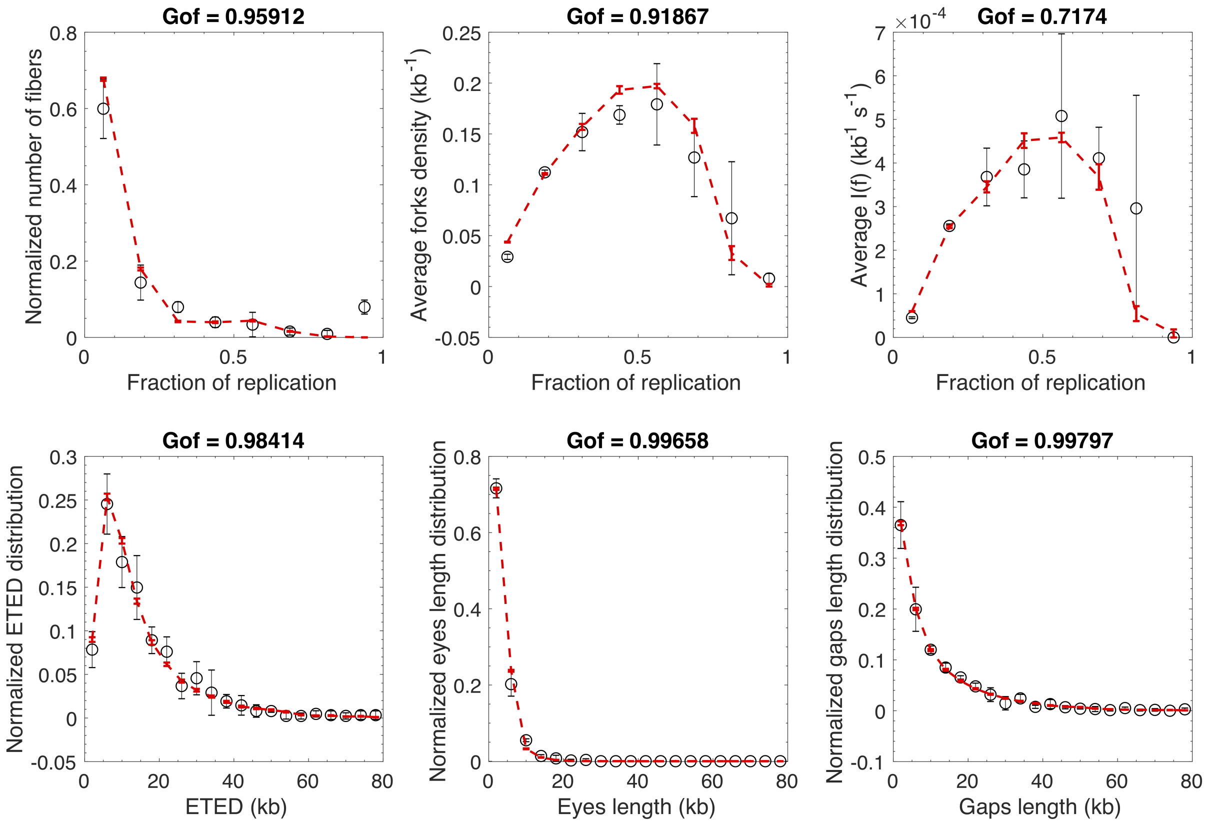

Figure S3. Modeling 53% global replicated fraction simulated data with discrete MM5 model. Open circles are simulated data and the red dashed line is the fit. $GoF_{global} = 0.82$

3 Reduction of MM6 to MM5

In the MM6 model origins fire globally with two origin firing probabilities (P_0 and $P_0 + P_{dom}$) eventually increased by a local origin firing probability (P_{fork}) close to an active fork, and the genome is divided into domains that either support or escape some inhibitory probability of firing (assumed to represent inhibition by the intra-S checkpoint). As the position of these domains is not identical between repeated simulations, we can reduce their description by specifying a fraction θ ($\theta = \frac{N_{dom} l_{dom}}{L}$) of the genome where origins escape checkpoint inhibition. In these domains, the global origin firing probability$P_{in} = \frac{1}{2}\left(P_0 + P_{dom}\right)$, with the $\frac{1}{2}$ pre-factor being due to normalization considerations. The local probability of origin firing (close to a fork) inside a domain is $P_{local}^{in} = \frac{1}{2}\left(P_0 + P_{dom} + P_{fork}\right)$. Outside these domains, the global probability of origin firing is modulated by the probability of origin inhibition $P_{out} = \frac{1}{2} P_0 \left(1 - P_{inhib}\right)$. In the same manner the local probability of origin firing is modulated by the action of intra-S checkpoint and the local cancellation of inhibition process $P_{local}^{out} = \frac{1}{2}\left(P_0 + P_{fork}\right)\left[1 + P_{inhib}\left(P_{deinhib} - 1\right)\right]$. Local probabilities of origin firing only influence origins over a distance d_{fork} downstream of a fork. The MM5 model contains a unique local probability of origin firing, that corresponds to the average value of the two local probabilities of origin firing, $P_{local} = \theta P_{local}^{in} + \left(1 - \theta\right) P_{local}^{out}$. Therefore, by considering the essential ingredients of the MM6 model, we combined the parameters of the model to retrieve the parameters of MM5 (Table S2). The values of these

Table S2. Reducing MM6 to MM5.

MM5	equivalence with MM6
N_0	N_0
J (s^{-1})	J
θ	$\frac{N_{dom} l_{dom}}{L}$
P_{in}	$\frac{1}{2}\left(P_0 + P_{dom}\right)$
P_{local}	$\frac{1}{2}\left(P_0 + P_{fork}\right)\left[1 + \left(1 - \theta\right) P_{inhib}\left(P_{deinhib} - 1\right)\right] + \theta P_{dom}$
P_{out}	$\frac{1}{2} P_0 \left(1 - P_{inhib}\right)$
d (kb)	d

parameters can be compared directly to parameters of MM5 model obtained from the fitting of the simulated data for each sample (Table S3). To assess if the difference between the expected and the inferred value of a parameter is statistically significant we calculate $t = \frac{(\text{expected value} - \text{inferred value})^2}{\text{error}^2}$, for $t \geq 1$ the difference is statistically significant otherwise it is not. The values of parameters changed as the global replicated fraction increased (Figure S4 and Table S3). To assess the level of significance of these variations we calculated $\chi^2 = \frac{(\text{parameter}_1 - \text{parameter}_2)^2}{\text{error}_1^2 + \text{error}_2^2}$ coefficient between the values of the same parameter obtained for different global replicated fraction. If $\chi^2 < 1$ the difference between the two values was not statistically significant otherwise it was significant. Figure S5 shows that the differences of predicted parameters values among the 3 considered samples were not statistically significant, as was expected. All $t < 1$ and $\chi^2 < 1$ (Figure S5), meaning the constancy of parameters values for all three samples. Therefore, we conclude that the optimization procedure was able to circumscribe the expected parameters values in an accurate manner for each sample. It should be noted that we choose a very conservative criterion to assess if two parameters are different or not. The conditions of $\chi^2 = 1$ or $t = 1$ are equivalent to a confidence level of $\alpha = 10^{-7}$ in the case of a two sided and one sided t statistics. In other words, with our criterion the probability to find that the values of two parameters are different by chance is smaller than 10^{-7}.

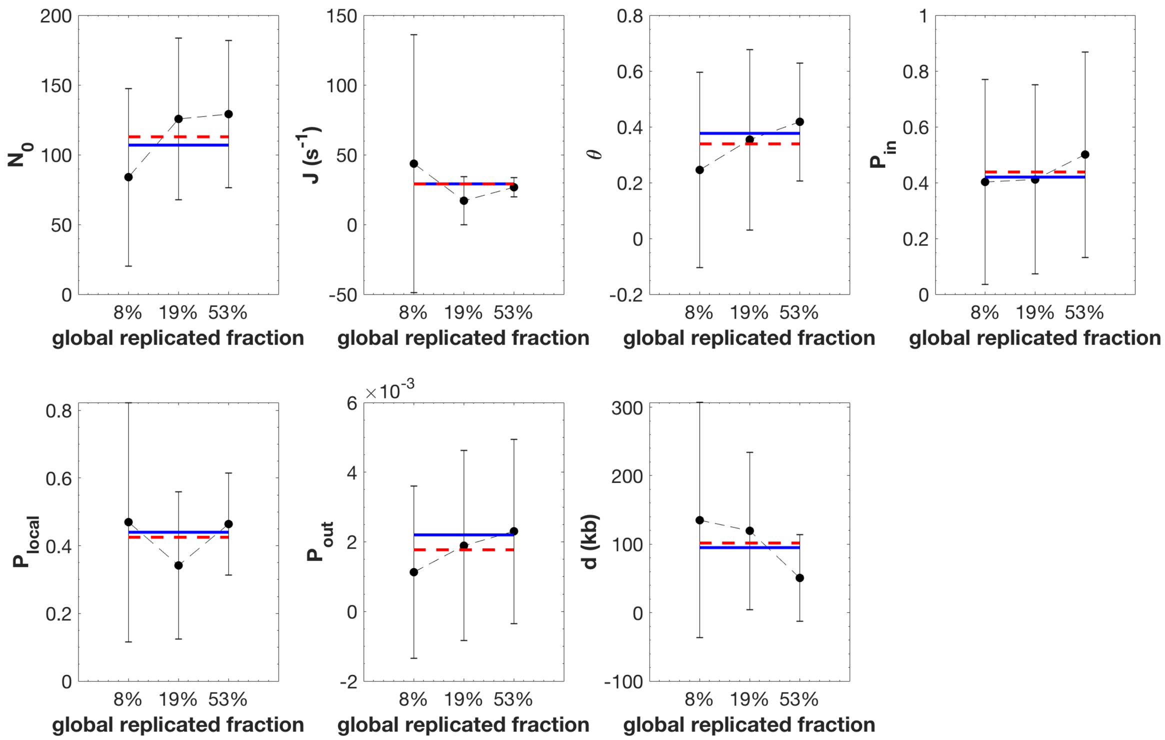

Figure S4. The fitting strategy infers accurately the expected values for the reduced MM6 free parameters. The black circles correspond to the averaged value of the parameter over 100 independent fits and the error bars are the standard-deviations. The solid blue line is the expected value of the parameter as obtained in Table S3. The red dashed line is the mean value of the parameter obtained by averaging the parameter inferred values over the 3 samples.

Table S3. Comparison between the expected and inferred values of MM5 parameters.

MM5	Input	8%	19%	53%
N_0	107	83.86 ± 32 ($t < 1$)	125 ± 29 ($t < 1$)	129 ± 26 ($t < 1$)
J (s^{-1})	29	43.6 ± 46 ($t < 1$)	17 ± 9 ($t < 1$)	27 ± 3.4 ($t < 1$)
θ	0.38	0.25 ± 0.2 ($t < 1$)	0.35 ± 0.16 ($t < 1$)	0.42 ± 0.1 ($t < 1$)
P_{in}	0.42	0.4 ± 0.2 ($t < 1$)	0.41 ± 0.17 ($t < 1$)	0.5 ± 0.2 ($t < 1$)
P_{local}	0.22	0.23 ± 0.09 ($t < 1$)	0.17 ± 0.05 ($t < 1$)	0.23 ± 0.04 ($t < 1$)
P_{out} ($\times 10^{-3}$)	2.2	1.1 ± 1 ($t < 1$)	1.9 ± 1 ($t < 1$)	2.3 ± 1 ($t < 1$)
d (kb)	94.91.	135 ± 86 ($t < 1$)	119 ± 57 ($t < 1$)	51 ± 32 ($t < 1$)

The ability of the fitting procedure i) to circumscribe the values of MM5 model parameters close to the expected ones (Table S2) and ii) to retrieve the constancy of these parameter's values as the global degree of replication increases (Figure S5) demonstrates the adequacy of our fitting strategy to recover the dynamic of DNA replication during S phase in the framework of MM5 model by setting the null hypothesis as : the values of MM5 parameters do not change as S phase progresses. Therefore, rejection of this hypothesis for a considered parameter means its variation during S phase.

N_0

	8%	19%	53%
8%	0.00	0.24	0.30
19%	0.24	0.00	0.00
53%	0.30	0.00	0.00

J (s^{-1})

	8%	19%	53%
8%	0.00	0.08	0.03
19%	0.08	0.00	0.27
53%	0.03	0.27	0.00

θ

	8%	19%	53%
8%	0.00	0.05	0.18
19%	0.05	0.00	0.03
53%	0.18	0.03	0.00

P_{in}

	8%	19%	53%
8%	0.00	0.00	0.03
19%	0.00	0.00	0.03
53%	0.03	0.03	0.00

P_{local}

	8%	19%	53%
8%	0.00	0.09	0.00
19%	0.09	0.00	0.21
53%	0.00	0.21	0.00

P_{out}

	8%	19%	53%
8%	0.00	0.04	0.10
19%	0.04	0.00	0.01
53%	0.10	0.01	0.00

d (kb)

	8%	19%	53%
8%	0.00	0.01	0.21
19%	0.01	0.00	0.27
53%	0.21	0.27	0.00

Figure S5. The values of each MM5 model parameter were compared pair-wise between samples with different global replicated fraction. The statistical significance of their difference was assessed by χ^2 test and represented as a binary heat map where not statistically significant differences are coloured in white and statistically significant difference are coloured in blue. The number in each box is the χ^2 coefficient.

In conclusion, any variation in parameter value detected by MM5 when analysing samples at different time points independently can be considered as statistically significant. Therefore, MM5 can adequately predict complex DNA replication dynamics using a limited number of processes.

References

1. McCune HJ, Danielson LS, Alvino GM, Collingwood D, Delrow JJ, Fangman WL, et al. The Temporal Program of Chromosome Replication: Genomewide Replication in clb5Δ Saccharomyces Cerevisiae. Genetics. 2008;180(4):1833–1847. doi:10.1534/genetics.108.094359.

2. Yang SCH, Rhind N, Bechhoefer J. Modeling Genome-Wide Replication Kinetics Reveals a Mechanism for Regulation of Replication Timing. Molecular Systems Biology. 2010;6(1):404. doi:10.1038/msb.2010.61.

3. Rhind N, Gilbert DM. DNA Replication Timing. Cold Spring Harb Perspect Biol. 2013;5(8):a010132. doi:10.1101/cshperspect.a010132.

4. Boulos RE, Drillon G, Argoul F, Arneodo A, Audit B. Structural Organization of Human Replication Timing Domains. FEBS Letters. 2015;589(20, Part A):2944–2957. doi:10.1016/j.febslet.2015.04.015.

5. Das SP, Borrman T, Liu VWT, Yang SCH, Bechhoefer J, Rhind N. Replication Timing Is Regulated by the Number of MCMs Loaded at Origins. Genome Res. 2015;25(12):1886–1892. doi:10.1101/gr.195305.115.

6. Petryk N, Kahli M, d'Aubenton-Carafa Y, Jaszczyszyn Y, Shen Y, Silvain M, et al. Replication Landscape of the Human Genome. Nat Commun. 2016;7(1):10208. doi:10.1038/ncomms10208.

7. Siefert JC, Georgescu C, Wren JD, Koren A, Sansam CL. DNA Replication Timing during Development Anticipates Transcriptional Programs and Parallels Enhancer Activation. Genome Res. 2017;27(8):1406–1416. doi:10.1101/gr.218602.116.

8. Platel M, Goldar A, Wiggins JM, Barbosa P, Libeau P, Priam P, et al. Tight Chk1 Levels Control Replication Cluster Activation in Xenopus. PLoS One. 2015;10(6). doi:10.1371/journal.pone.0129090.
