# Supplementary material for: Organization of DNA Replication Origin Firing in Xenopus Egg Extracts: The Role of Intra-S Checkpoint
Source: Genes (Basel). 2021 Aug 9;12(8):1224. doi: 10.3390/genes12081224 (PMC8394201; doi:10.3390/genes12081224)
Supplement: Supplementary file 1 [file genes-12-01224-s001.zip › S3.pdf]

Supplementary Material S3

1 Fitting the experimental profiles by MM5 model : Unchallenged S phase

We fitted independently the measured profiles for each global replicated fraction by discrete MM5 model. The fits of observations from 8% global replicated fraction are presented in Appendix 1, Figure 9 and those of 19% and 53% are presented in Figures S1 and S2 respectively. In Table S1 we give the value of the fitted parameters. The reliability of observed differences among inferred MM5 parameters are assessed statistically by using χ^2 coefficient as defined in Supplementary Material S2 (Figure S3).

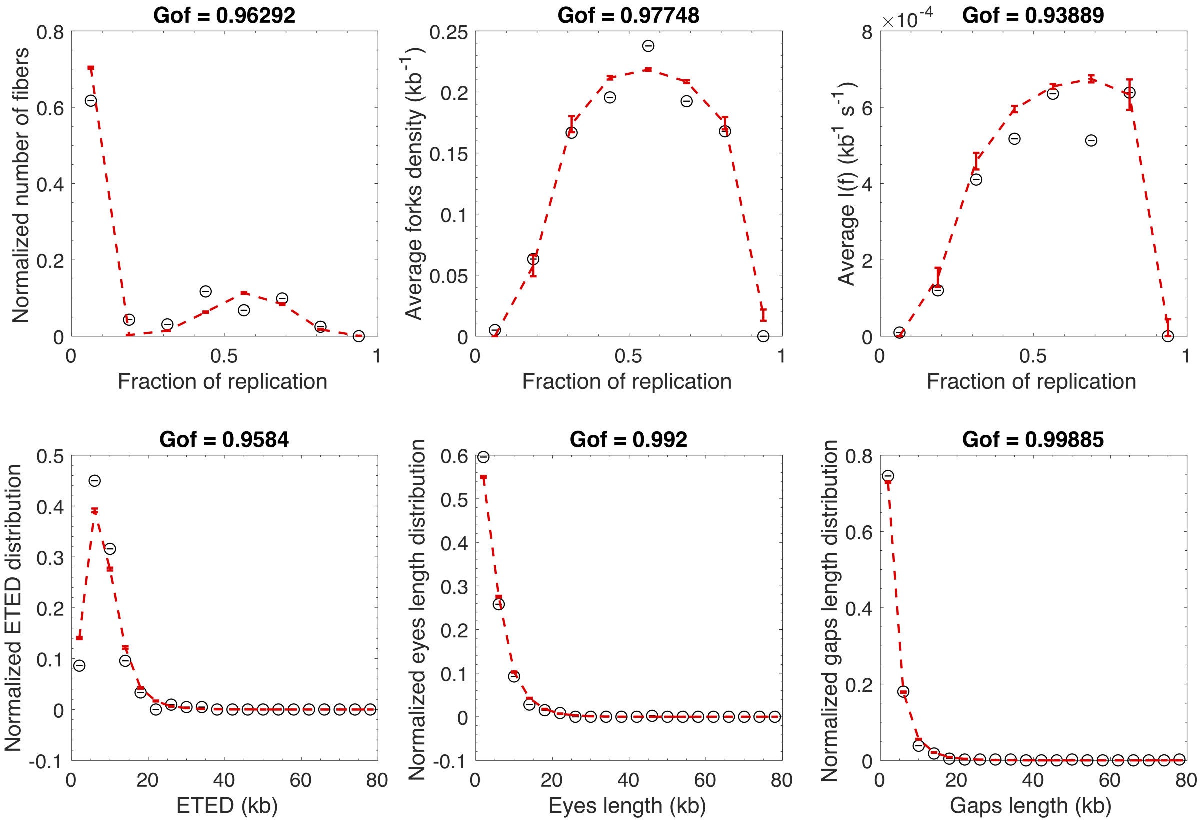

Figure S1. Modeling measured sample with 19% global replicated fraction with the discrete MM5 model. Open circles are simulated data and the red dashed line is the fit. $GoF_{global} = 0.96$

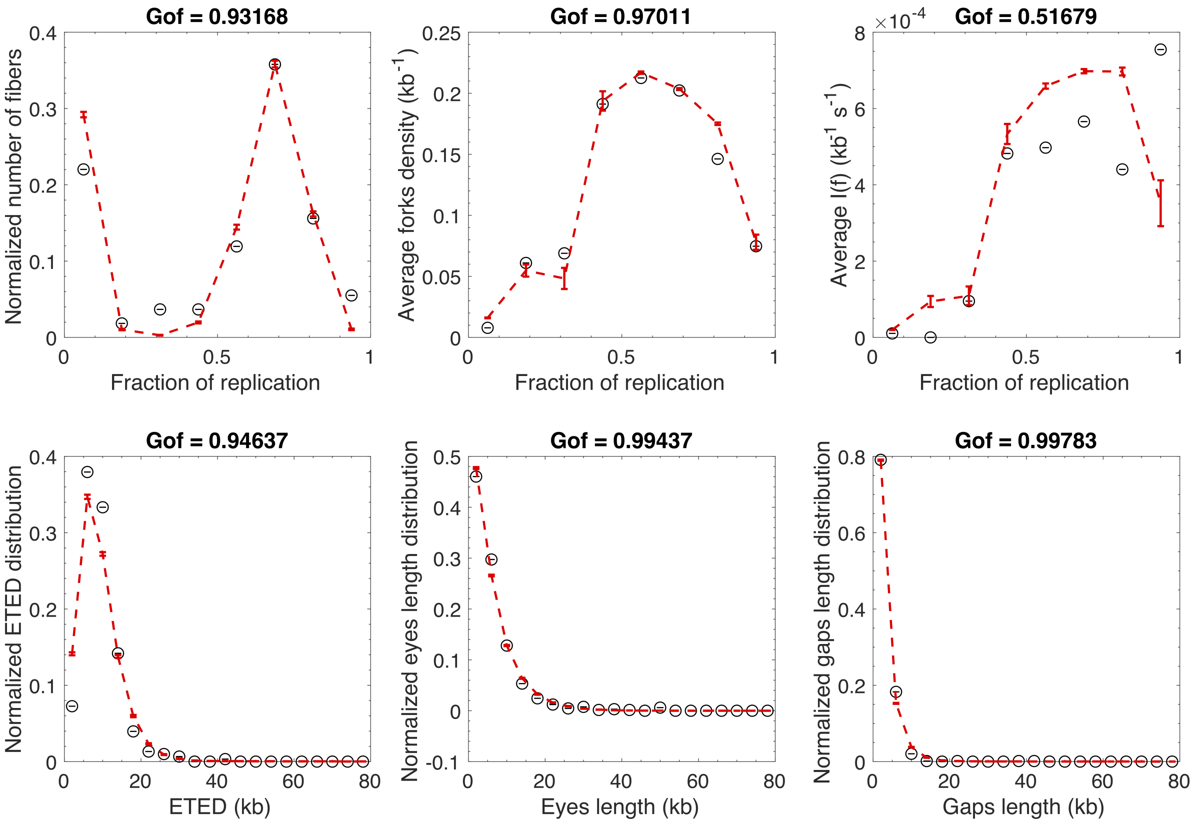

Figure S2. Modeling measured sample with 53% global replicated fraction with the discrete MM5 model. Open circles are simulated data and the red dashed line is the fit. $GoF_{global} = 0.90$

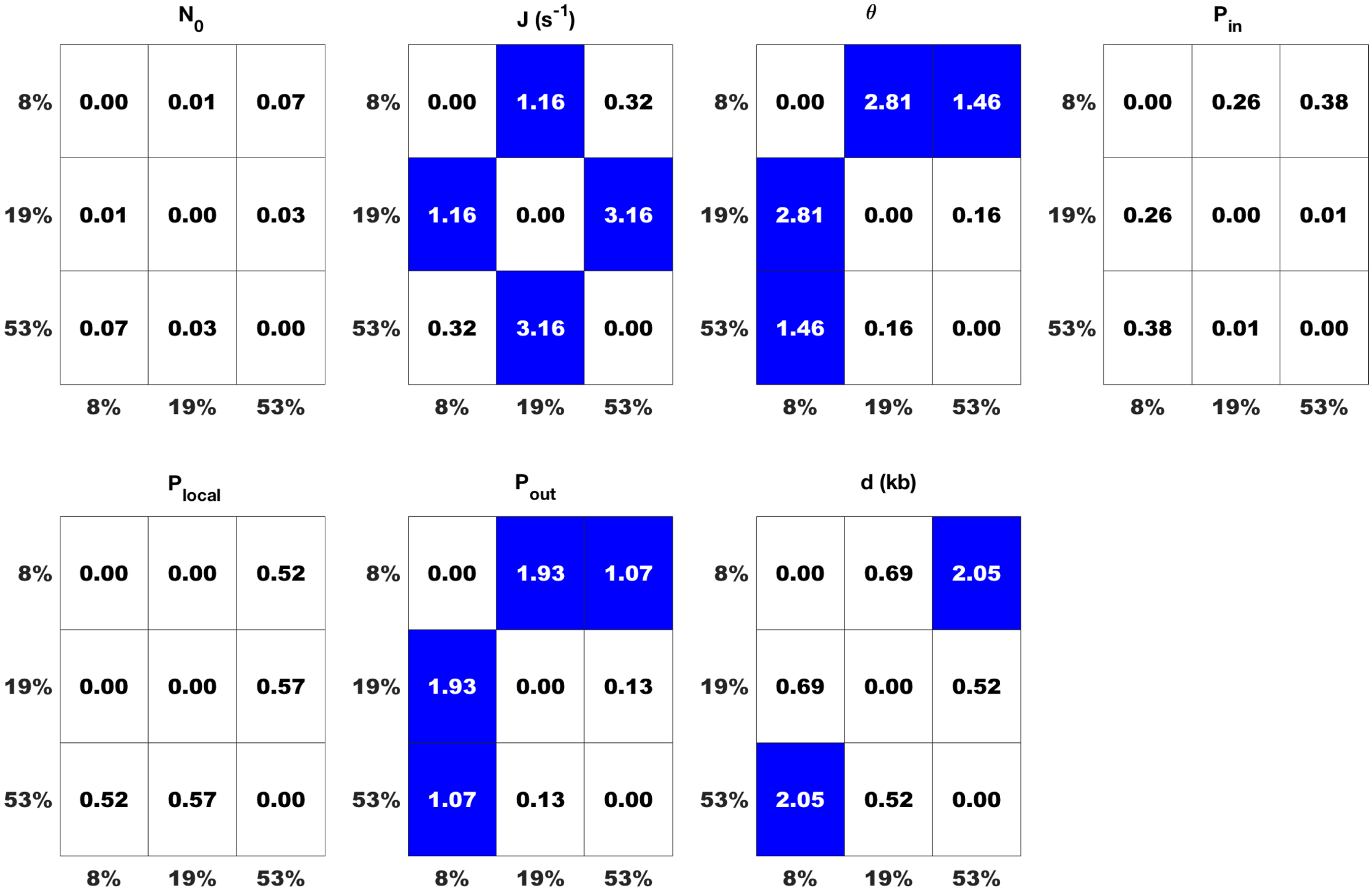

Figure S3. The values of each MM5 model parameter were compared pair-wise between samples with different global replicated fraction. The statistical significance of their difference was assessed by χ^2 test and represented as a binary heat map where the white colour represents no statistically significant difference and the blue colour represents statistically significant difference. The number in each box is the χ^2 coefficient.

2 Fitting the experimental profiles by MM5 model : Chk1 inhibited S phase

We fitted with the discrete MM5 model a sample that had spent in the presence of UCN-01 the same time interval in S phase as the control sample with 8% global replicated fraction. The global replicated fraction of the of the UCN-01 sample was 22%. The fits are presented in Figure S4 and the obtained parameters values are given in Table S1. The reliability of observed differences among inferred MM5 parameters between controls and Chk1 inhibited sample are assessed statistically by using χ^2 coefficient as defined in Supplementary Material S2 (Figure S5).

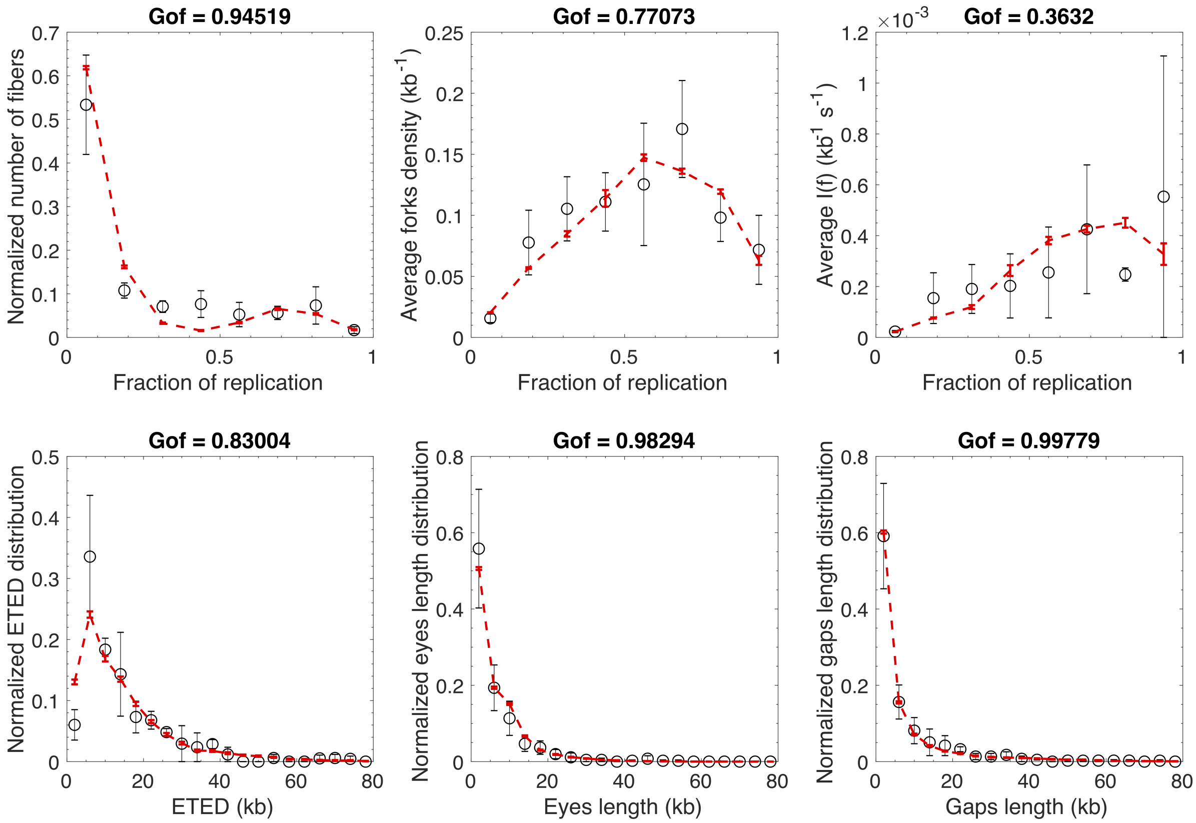

Figure S4. Modeling a measured sample with 22% global replicated fraction in presence of UCN-01with discrete MM5 model. Open circles are simulated data and the red dashed line is the fit. $GoF_{global} = 0.85$

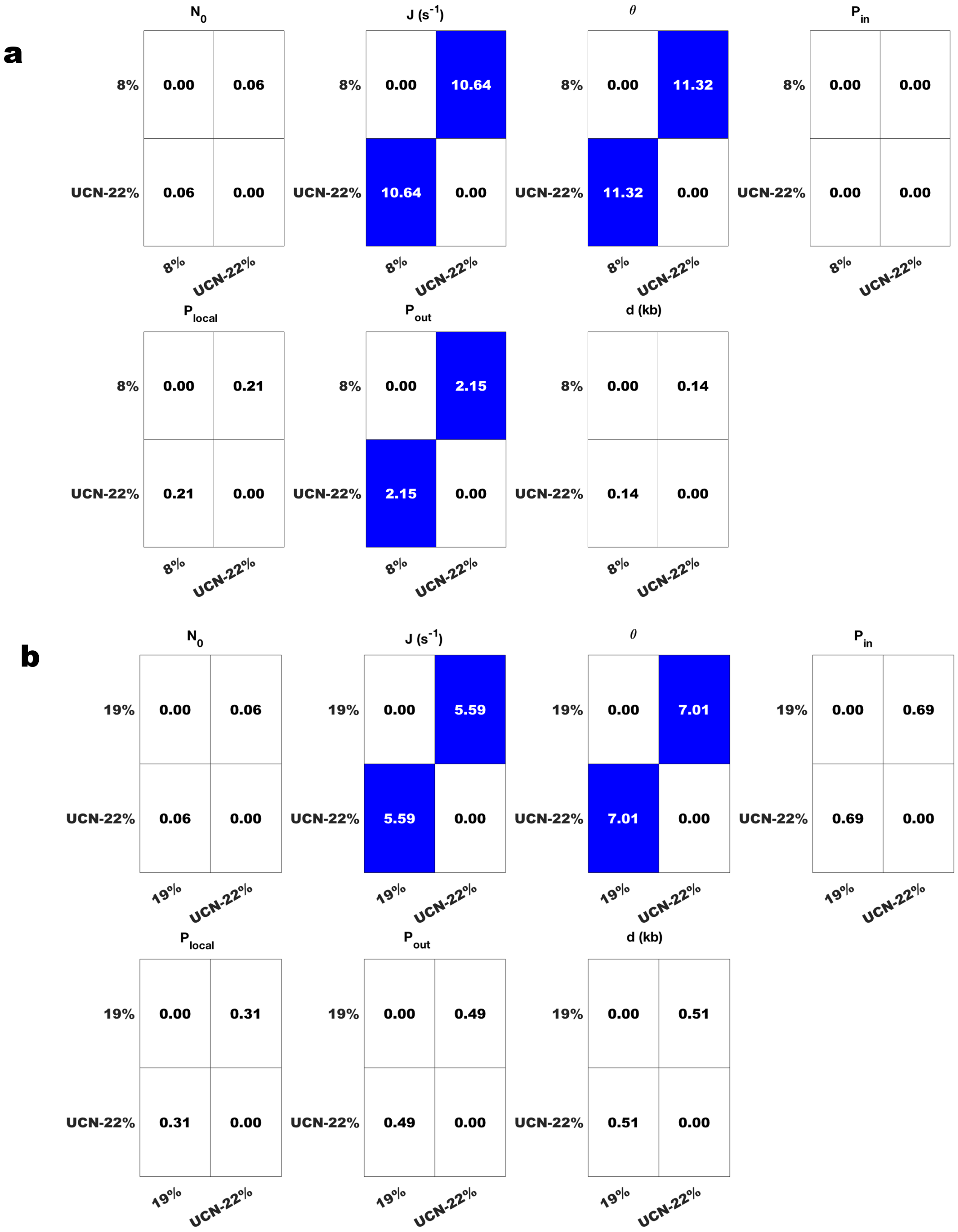

Figure S5. a. Comparing samples that have spent the same time interval in S phase. b. Comparing samples that have similar global replication fractions. The values of each MM5 model parameter were compared pair-wise between samples with different global replicated fraction. The statistical significance of their difference was assessed by χ^2 test and represented as a binary heat map where the white colour represents no statistically significant difference and the blue colour represents statistically significant difference. The number in each box is the χ^2 coefficient.

3 Fitting the experimental profiles by MM5 model : Chk1 over-expressed S phase

We fitted with the discrete MM5 model a Chk1 over-expressed sample with the same incubation time than the control sample with 46% global replicated fraction. The global replicated fraction of the of the Chk1 over-expressed sample was 22%. The fits are presented in Figures S6 and S7 and the obtained parameters values are given in Table S1. The reliability of observed differences among inferred MM5 parameters between controls and Chk1 over-expressed sample are assessed statistically by using χ^2 coefficient as defined in Supplementary Material S2 (Figure S8).

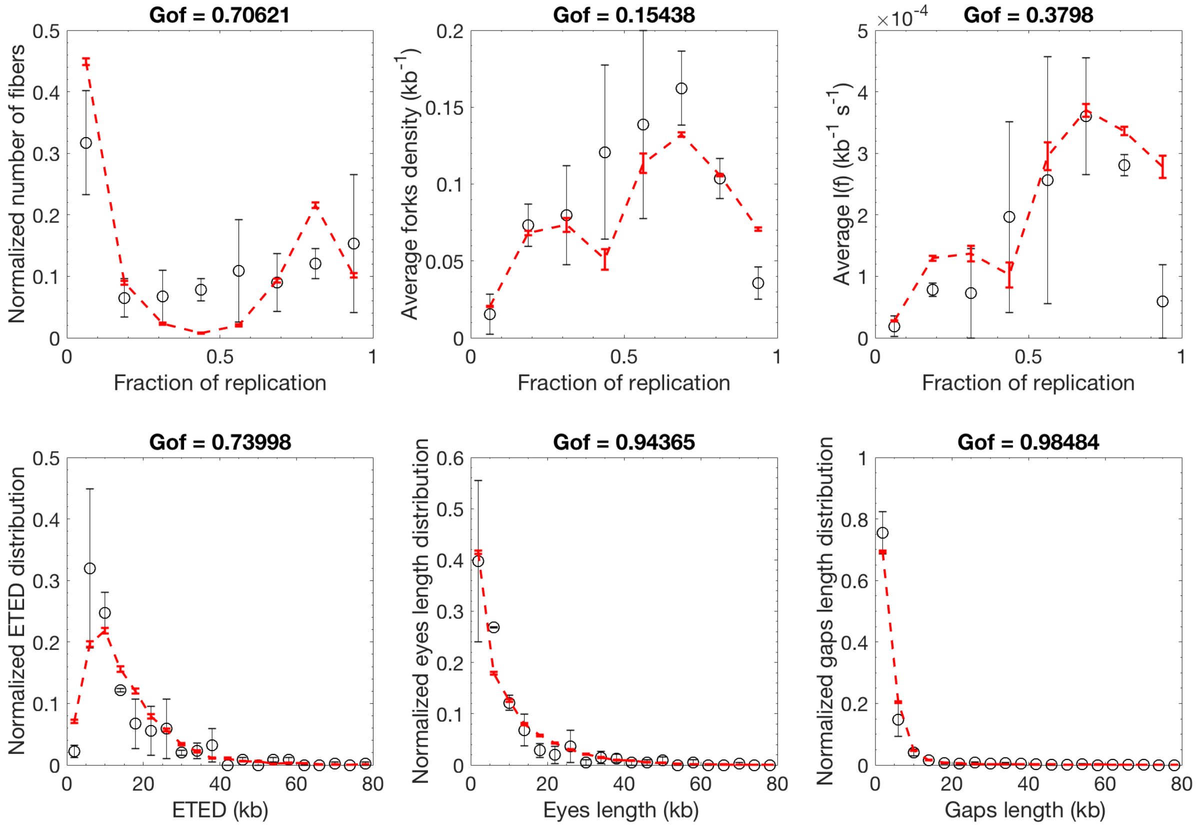

Figure S6. Modeling a measured sample with 46% global replicated fraction with discrete MM5 model. Open circles are simulated data and the red dashed line is the fit. $GoF_{global} = 0.65$

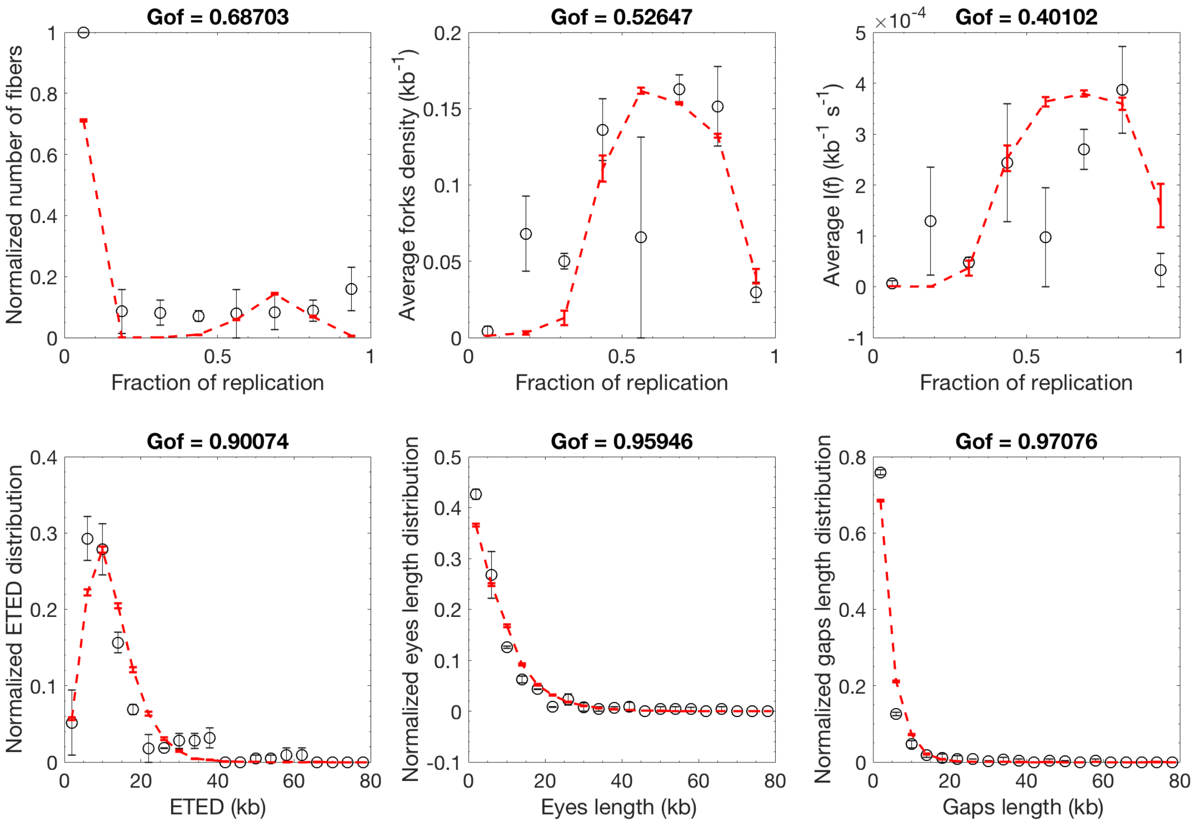

Figure S7. Modeling a measured sample with 22% global replicated fraction where Chk1 is over-expressed with discrete MM5 model. Open circles are simulated data and the red dashed line is the fit. $GoF_{global} = 0.74$

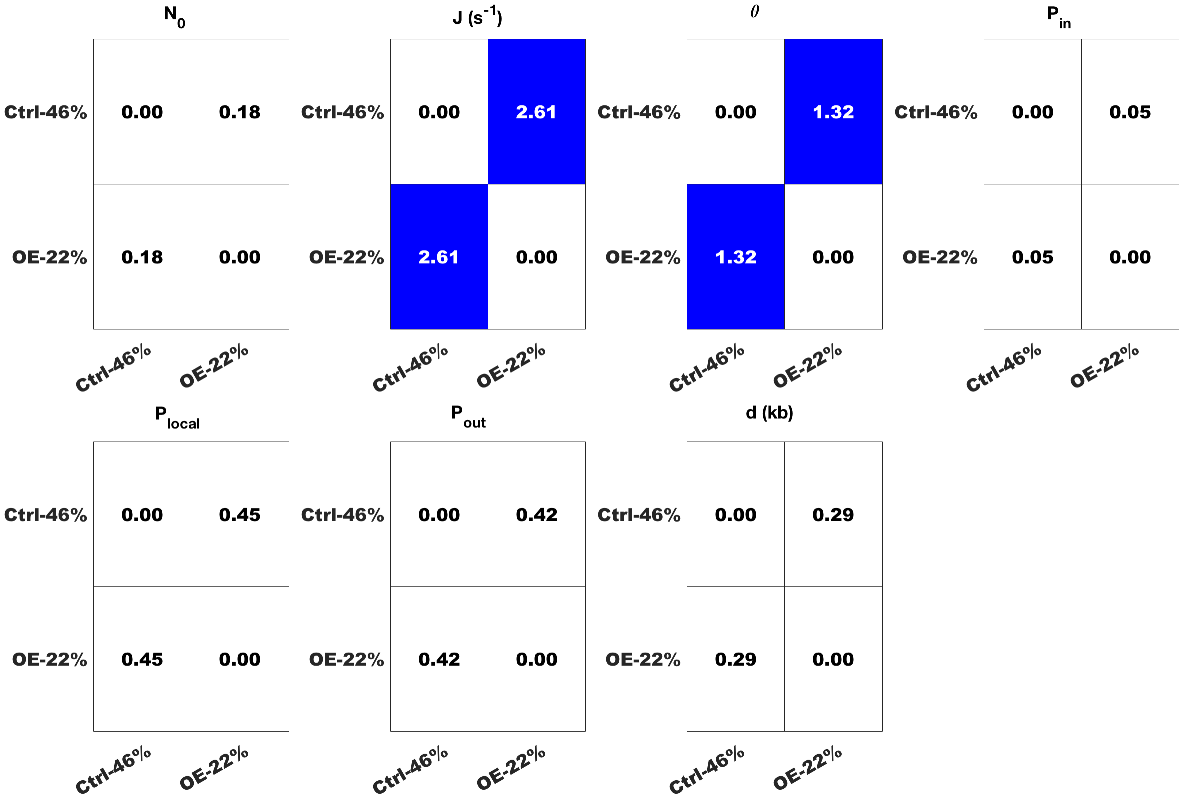

Figure S8. Comparing samples that have spent the same time interval in S phase. The values of each MM5 model parameter were compared pair-wise between samples with different global replicated fraction. The statistical significance of their difference was assessed by χ^2 test and represented as a binary heat map where the white colour represents no statistically significant difference and the blue colour represents statistically significant difference. The number in each box is the χ^2 coefficient.

Table S1. Values and the corresponding errors of MM5 parameters for the best fit of each sample and each condition.

MM5	unchallenged: 8%	unchallenged: 19%	unchallenged: 53%	UCN-01: 22%	unchallenged: 46%	over-expressed: 22%
N_0	1064 ± 135	1043 ± 116	1002 ± 106	1006 ±102	880±58.5	784±58.7
J (s^{-1})	601 ± 198	1026 ± 196	404 ± 151	1467 ± 89	1190±114	569±82.1
θ	0.25 ± 0.06	0.43 ± 0.04	0.39 ± 0.05	0.56 ± 0.032	0.50±0.01	0.42±0.02
P_{in}	0.41 ± 0.07	0.34 ± 0.07	0.32 ± 0.07	0.42 ± 0.07	0.33±0.03	0.36±0.04
P_{local}	0.43 ± 0.06	0.43 ± 0.06	0.52 ± 0.06	0.38 ± 0.06	0.37±0.03	0.45±0.03
P_{out}	0.09± 0.02	0.17 ± 0.04	0.15 ± 0.03	0.23 ± 0.04	0.15±0.02	0.23±0.03
d (kb)	143.8 ± 36.3	91.5 ± 25.6	56.1 ± 23.6	119.3 ± 29.3	139.9±16.9	178.4±18.7
