# Supplementary material for: Organization of DNA Replication Origin Firing in Xenopus Egg Extracts: The Role of Intra-S Checkpoint
Source: Genes (Basel). 2021 Aug 9;12(8):1224. doi: 10.3390/genes12081224 (PMC8394201; doi:10.3390/genes12081224)
Supplement: Supplementary file 1 [file genes-12-01224-s001.zip › S1.pdf]

Supplementary Material S1

# 1 Different models

To model experimental observations a series of nested models (Table S1) were compared with experimental data. Below are the fits of each model to experimental sample with

**Table S1. The nested models and their variables**

| Model | $N_0$ | $J$ | $P_{out}$ | $P_{in}$ | $P_{local}$ | $\theta$ | $d$ | $v$ |
|---|---|---|---|---|---|---|---|---|
| MM1 | √ | √ | √ | | | | | |
| MM2 | √ | √ | √ | | | | | √ |
| MM3 | √ | √ | √ | | √ | | √ | |
| MM4 | √ | √ | √ | √ | | √ | | |
| MM5 | √ | √ | √ | √ | √ | √ | √ | |

8% global replicated fraction. To assess the goodness of the fit (GoF) we considered the normalised mean square error between the simulated profile and the fitted entity as the indicator of likelihood ($GoF = 1 - \frac{1}{N}\sum_{i=1}^{N}\frac{\left(y^i_{fit}-y^i_{exp}\right)^2}{\left(y^i_{exp}-\langle y_{exp}\rangle\right)^2}$, where $\langle y_{exp}\rangle$ represents average value of the experimental data set and $N$ is the number of data points ). GoF costs vary between $-\infty$ (bad fit) to 1 (perfect fit). If $GoF = 0$, $y_{fit}$ is no better than a straight line at matching experimental data. The global cost is calculated as $GoF_{global} = \frac{1}{6}\sum_{1}^{6} GoF_i$ where $i$ represents one fitted entity. All models reproduce with the same accuracy the distribution of replicated fibres, gaps lengths and eyes lengths distributions. The major contributions to score values come from residuals of average fork density, average $I\,(f)$ and eye-to-eye distances distribution fits. From the value of $GoF_{global}$ (Table S2), the model that best described the whole data set is the MM5 with localized distribution of potential origins: its $GoF_{global}$ value is closest to one. However, MM5 also has the highest number of fitting variables (7) compared to other models ( MM1 has 3 fitting variables, MM2 has 4 fitting variables,MM3 and MM4 have 5 fitting variables), and facilitating fit to the data.

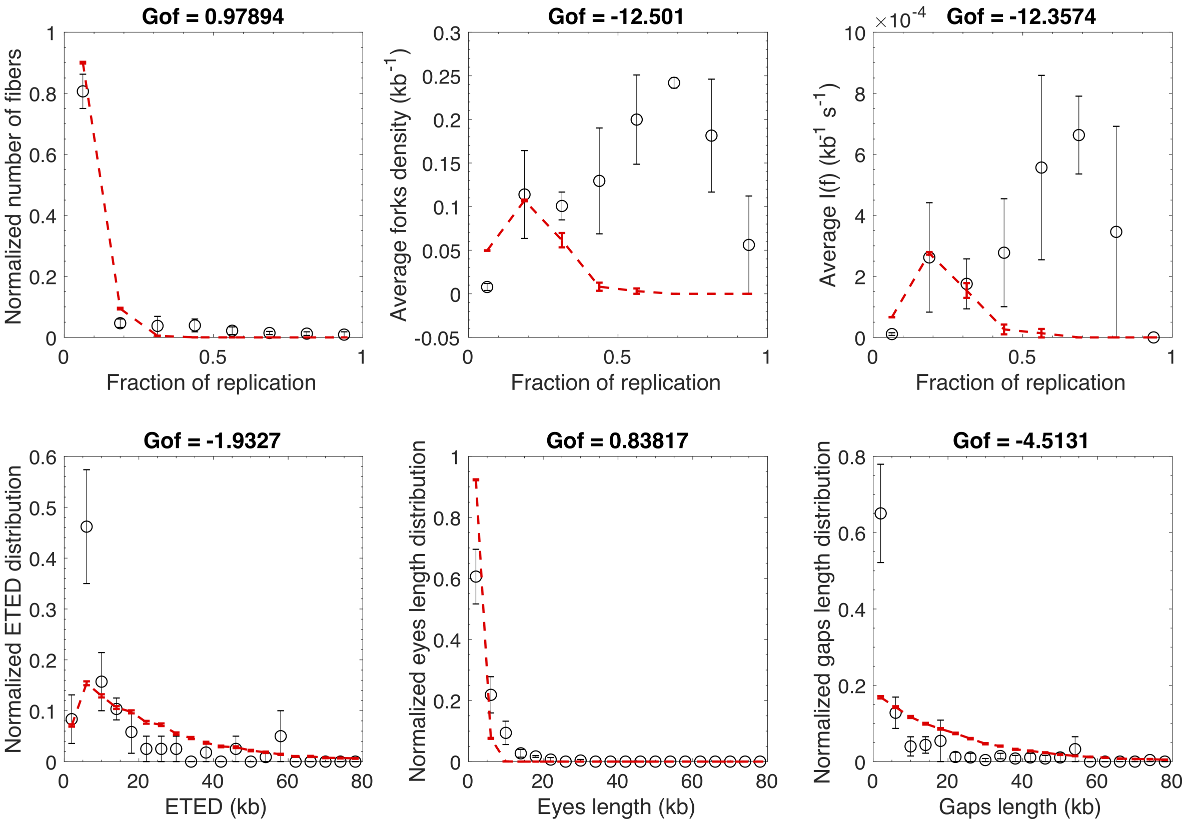


**Figure S1. Modeling experimental data with MM1 model in the case where the potential origins are continuously distributed along the genome.** Open circles are experimental data and the red dashed line is the fit.

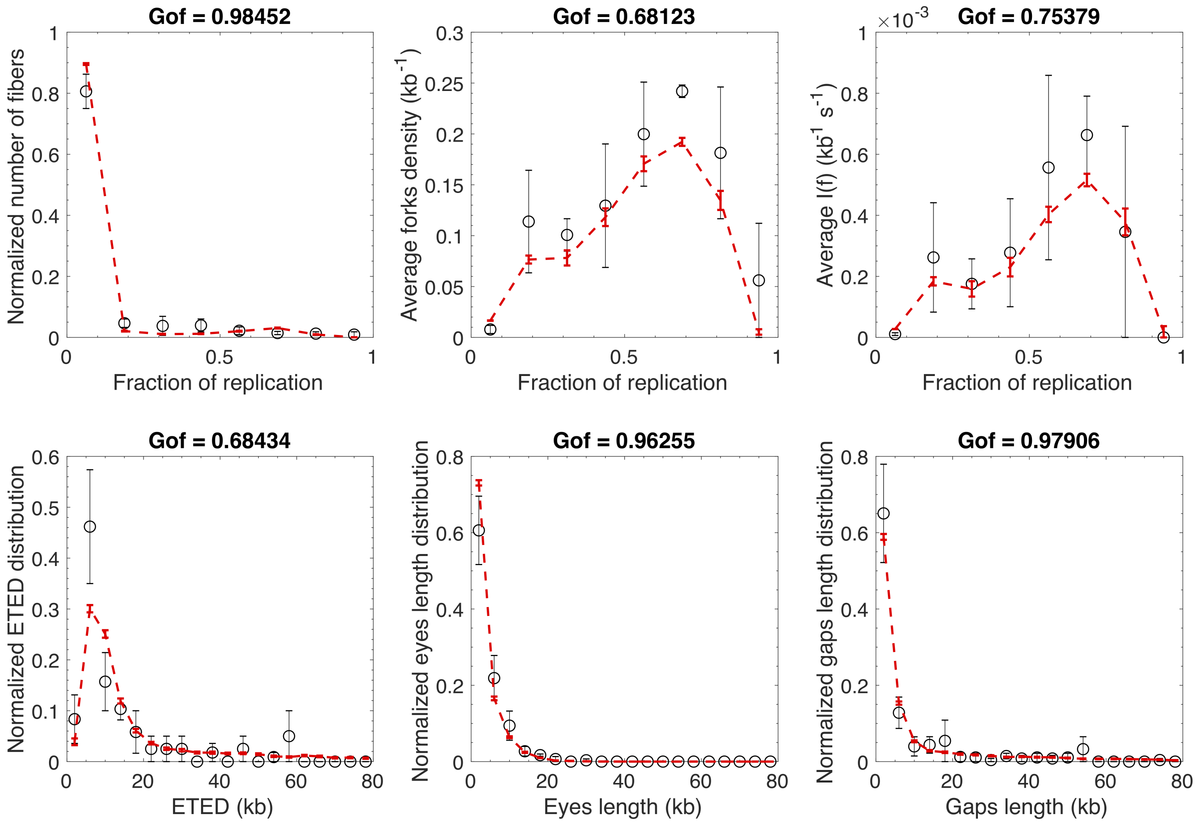


**Figure S2. Modeling experimental data with MM3 model in the case where the potential origins are continuously distributed along the genome.** Open circles are experimental data and the red dashed line is the fit.

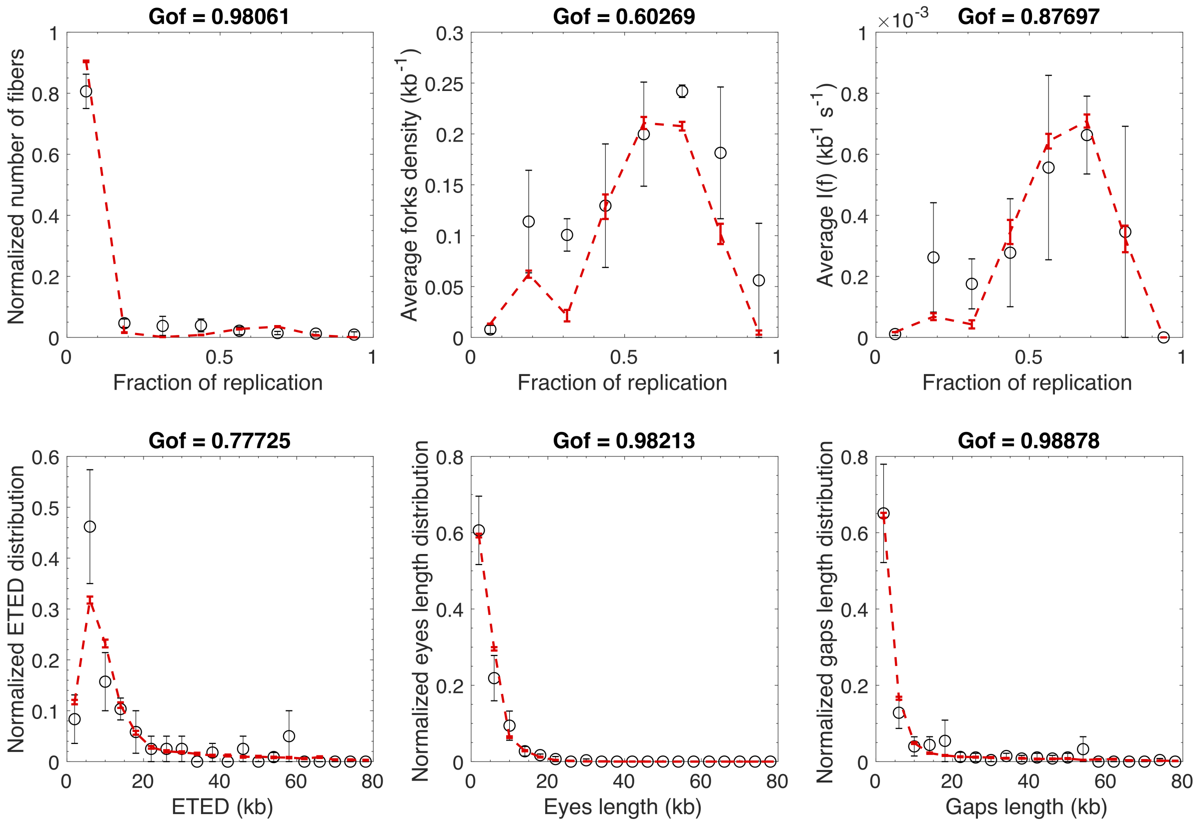


**Figure S3. Modeling experimental data with MM4 model in the case where the potential origins are continuously distributed along the genome.** Open circles are experimental data and the red dashed line is the fit.

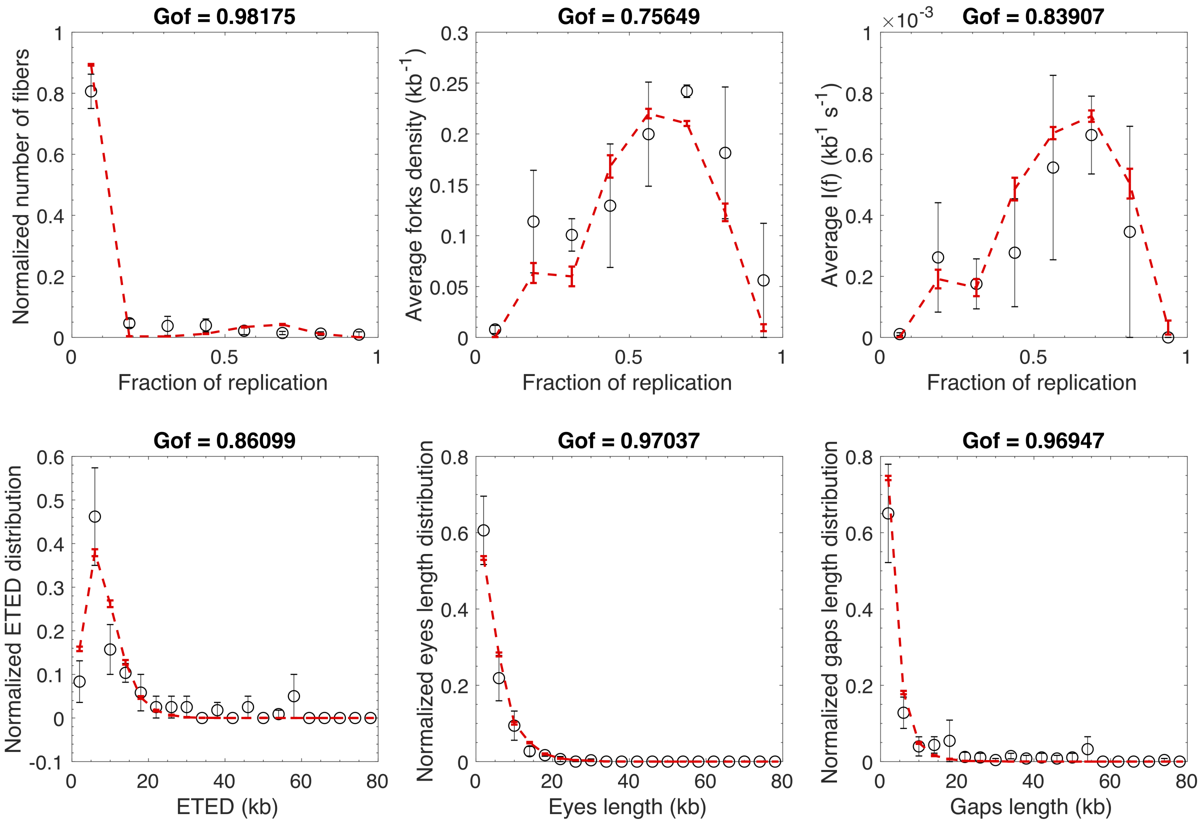


**Figure S4. Modeling experimental data with MM5 model in the case where the potential origins are continuously distributed along the genome.** Open circles are experimental data and the red dashed line is the fit.

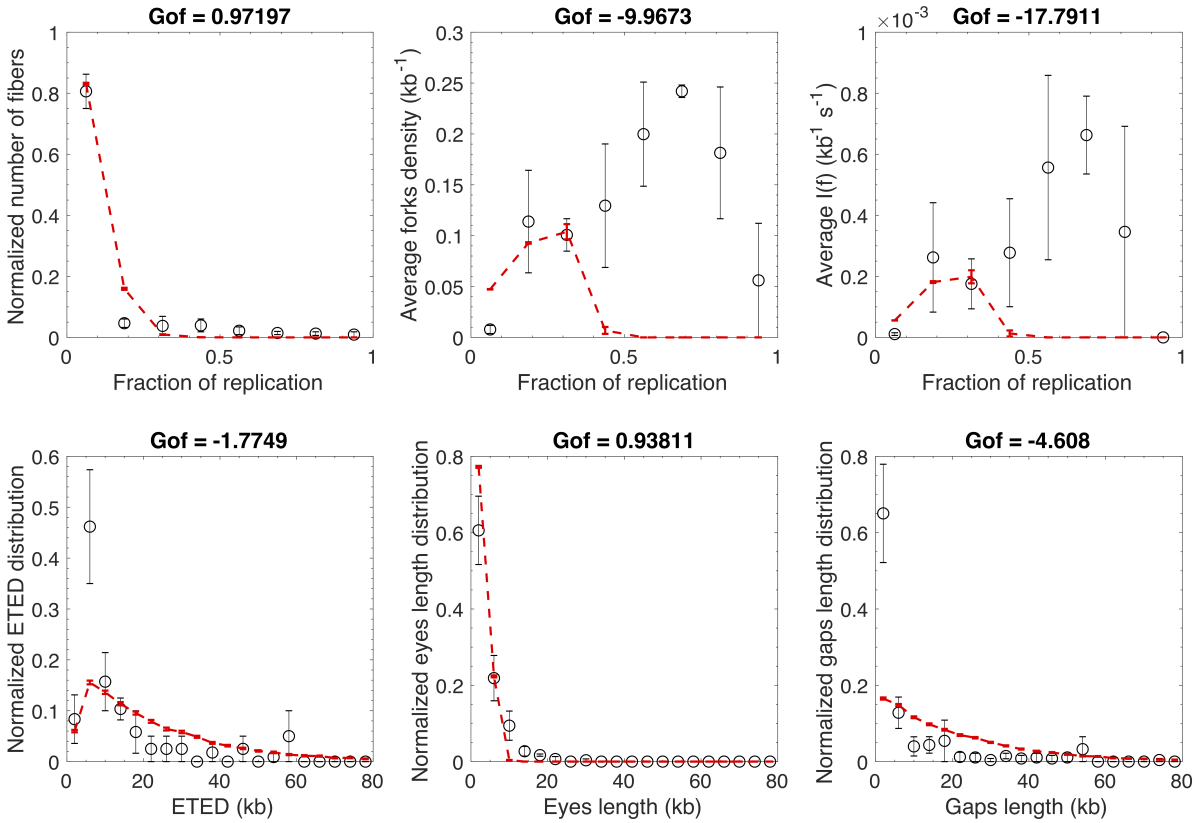


**Figure S5. Modeling experimental data with MM1 model in the case where the potential origins form a discrete set along the genome.** Open circles are experimental data and the red dashed line is the fit.

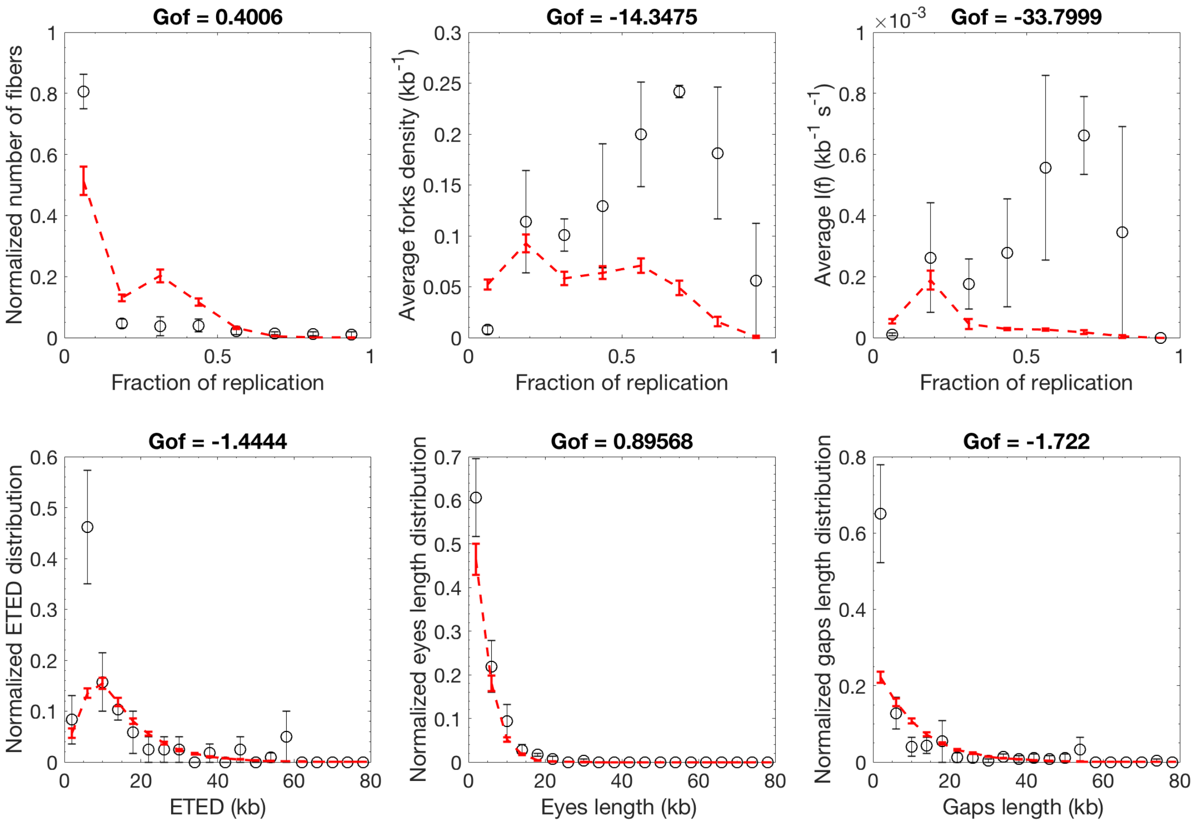


**Figure S6. Modeling experimental data with MM2 model in the case where the potential origins form a discrete set along the genome.** Open circles are experimental data and the red dashed line is the fit.

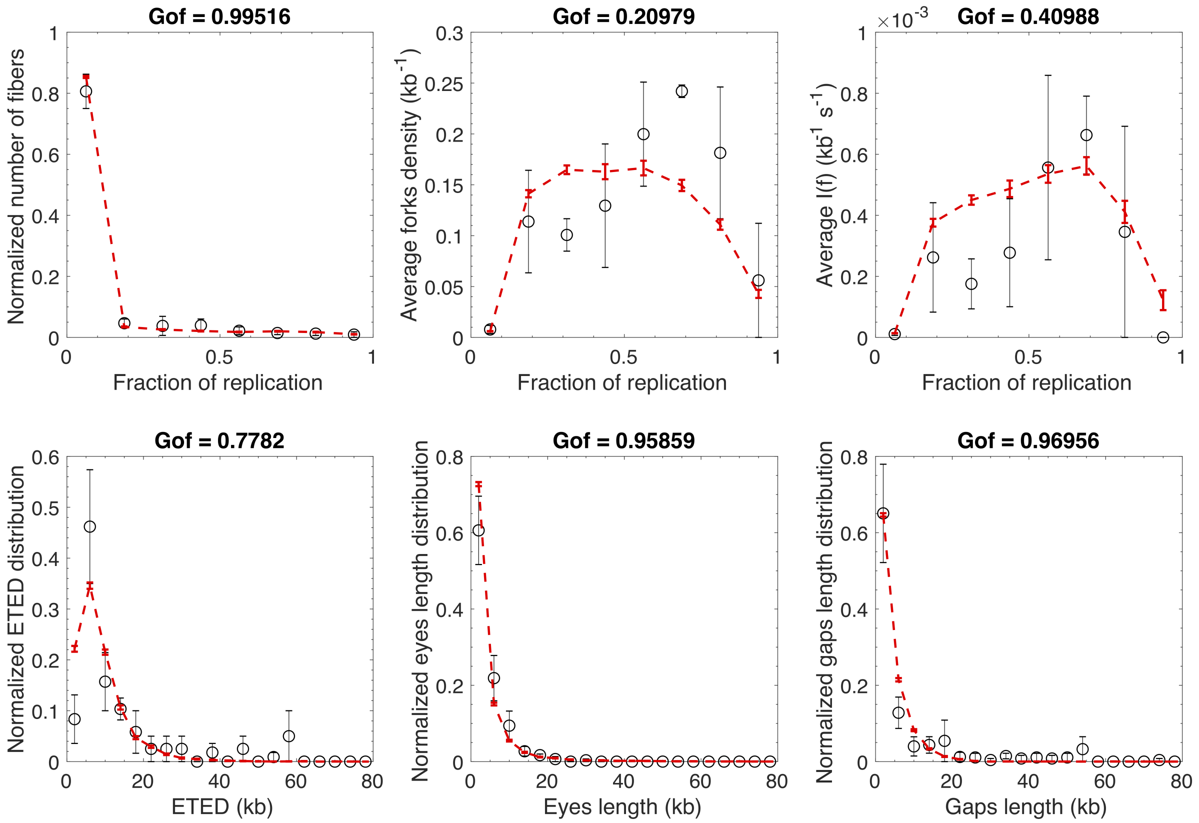


**Figure S7. Modeling experimental data with MM2 model in the case where the potential origins form a discrete set along the genome.** Open circles are experimental data and the red dashed line is the fit.

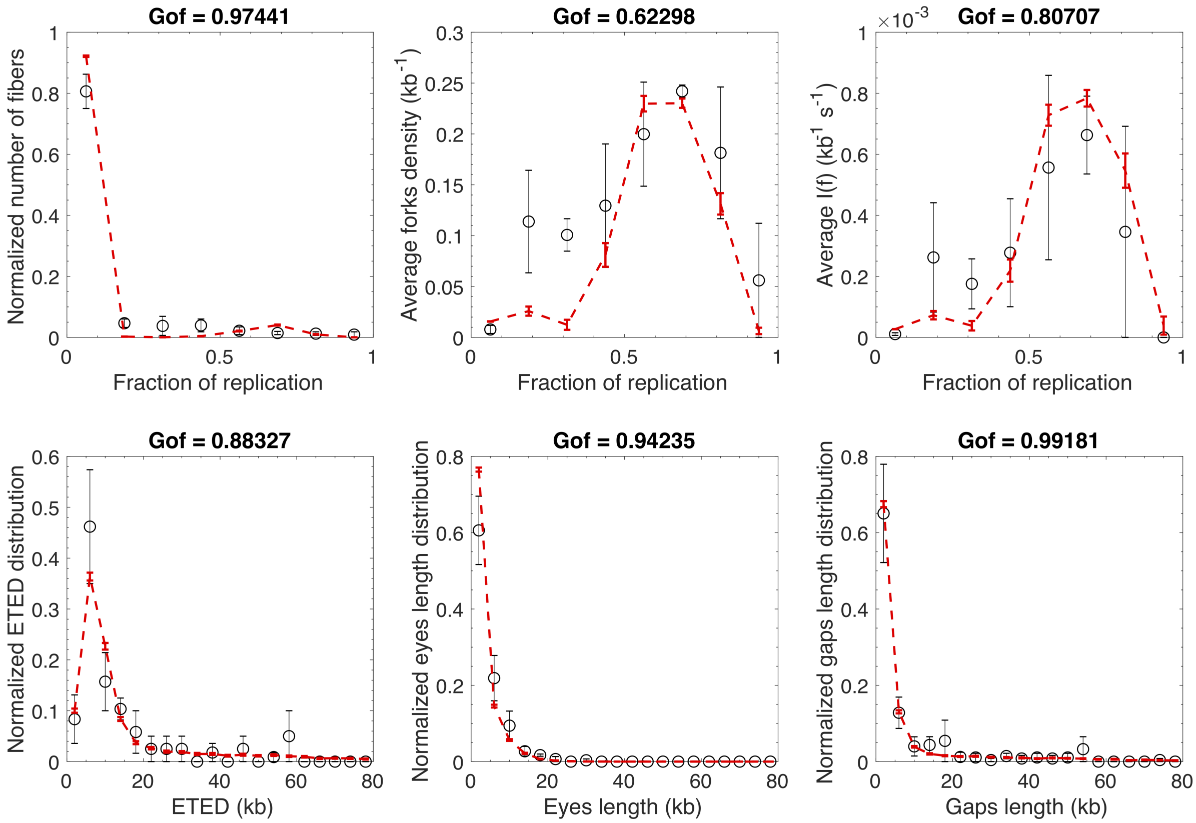


**Figure S8. Modeling experimental data with MM4 model in the case where the potential origins form a discrete set along the genome.** Open circles are experimental data and the red dashed line is the fit.

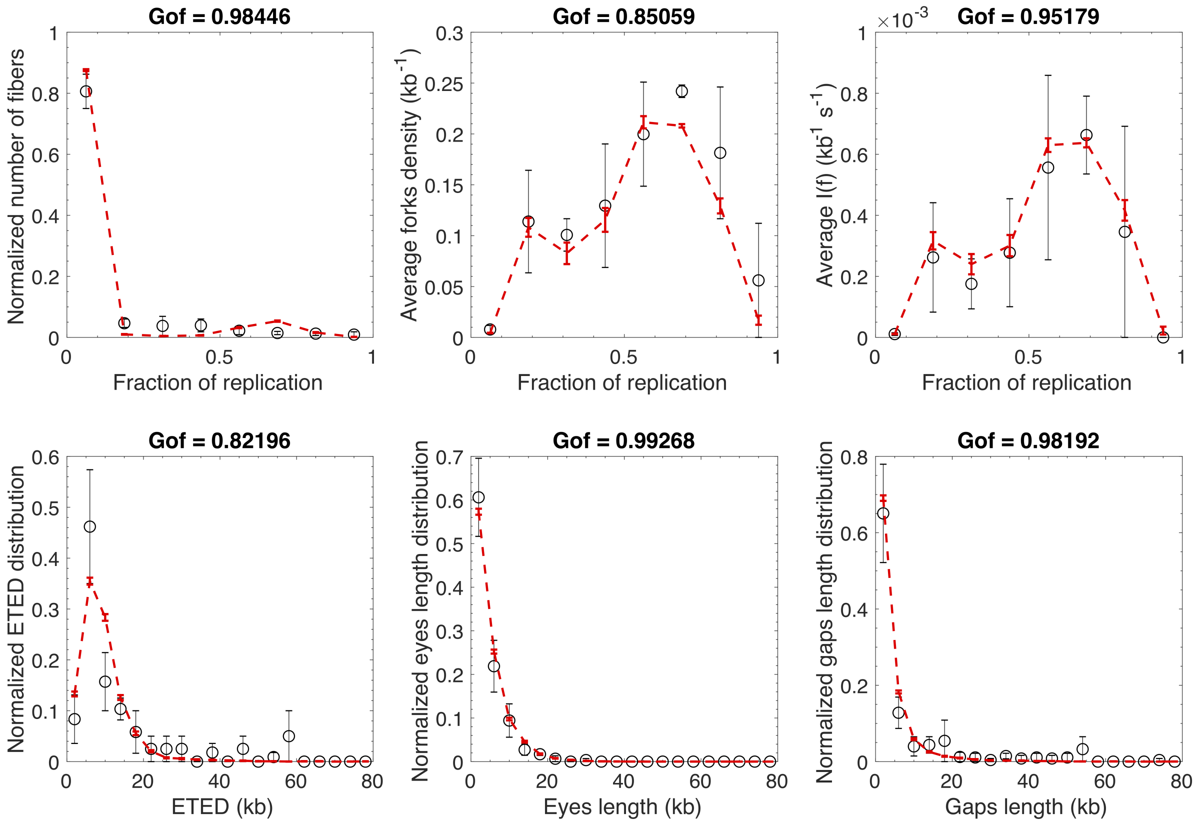


**Figure S9. Modeling experimental data with MM5 model in the case where the potential origins form a discrete set along the genome.** Open circles are experimental data and the red dashed line is the fit.

**Table S2. Values of $GoF_{global}$ and fitting residual norm ($(y_{exp} - y_{fit})^2$) for each model.**

| | Continuous | Discrete | Continuous | Discrete |
|---|---|---|---|---|
| model | $GoF_{global}$ | $GoF_{global}$ | $(y_{exp} - y_{fit})^2$ | $(y_{exp} - y_{fit})^2$ |
| MM1 | -0.95 | -5.28 | 0.66 | 0.56 |
| MM2 | | -8.34 | | 0.53 |
| MM3 | 0.85 | 0.72 | 0.08 | 0.10 |
| MM4 | 0.87 | 0.88 | 0.08 | 0.09 |
| MM5 | 0.90 | 0.92 | 0.08 | 0.05 |

## 2 Models comparison

To address whether the better data fit with MM5 is solely due to the higher degree of complexity of the model, we used two different approaches : a traditional statistical hypothesis testing: the extra sum of squares F test [1] and the Akaike's criterion ($\Delta AIC$) that is based on information theory [2]. We can objectively reject MM1 and MM2 as they did not reproduce in a satisfactory manner the averaged fork density, $I(f)$ and eye-to-eye distances distributions (Figures S1, S5 and S6). MM3 and MM4 satisfactorily reproduced all measured quantities (Figures S2 , S3, S7 and S8 ) but with lower $GoF_{global}$ value than the MM5 models (Table S2). The discrete MM5 model has higher $GoF_{global}$ value than the continuous one, whereas the continuous MM3 and MM4 models were better than or equal to their discrete version, respectively (Table S2). To choose the best model, we compared the discrete MM5 model, continuous MM3, MM4 and MM5 corresponding to fits with highest $GoF_{global}$ values (Table S2). Comparing the discrete MM5 with the continuous MM3, MM4 and MM5 models led in all cases to $F > 1$ with p-values $p < 10^{-6}$ and negative $\Delta AIC$ values (Table S3). To verify if the increase in model's complexity does always leads to $F > 1$ and negative $\Delta AIC$ values, we fitted the experimental data with the MM6 model as described in Supplementary Material S2 (Figure S10, $GoF_{global} = 0.87$ and $(y_{exp} - y_{fit})^2 = 0.07$) that has 10 adjustable parameters.

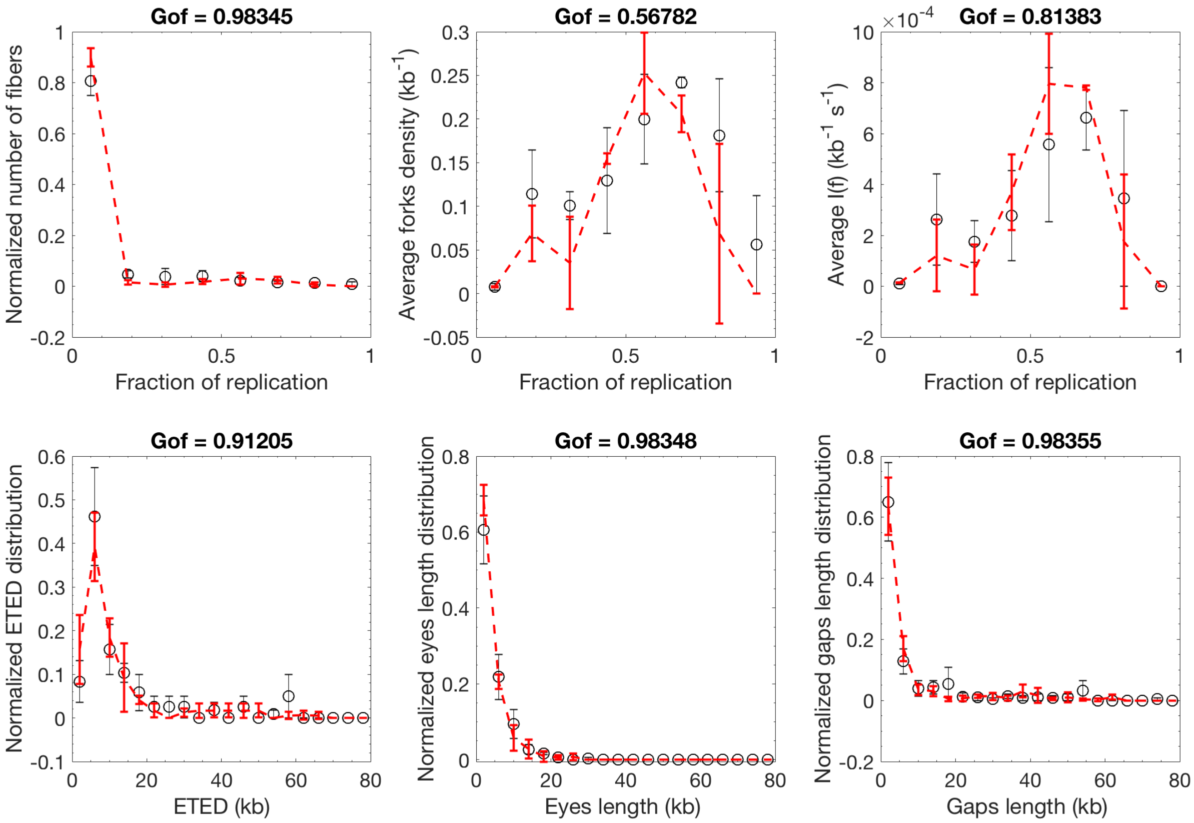


**Figure S10. Modeling experimental data with MM6 model in the case where the potential origins form a discrete set along the genome.** Open circles are experimental data and the red dashed line is the fit.

In this case $F < 1$ and $\Delta AIC > 0$ (Table S3), implying that MM6 is overfitting the data. The discrete MM5 model is therefore the best model and the observed increase in $GoF_{global}$ is not the consequence of increased complexity of the model.

**Table S3. Values of F-test, the associated *p*-value ($p$) and the $\Delta AIC$ when the discrete MM5 model is compared with continuous MM3, MM4 and MM5 model.**

| model | $F$ | $p$ | $\Delta AIC$ |
|---|---|---|---|
| Continuous MM3 | 19.3 | $1.5\times10^{-7}$ | -30.2 |
| Continuous MM4 | 16.9 | $8.3\times10^{-7}$ | -26.6 |
| Continuous MM5 | $\infty$ | Not defined | -31.1 |
| MM6 | $-5.3$ | 1 | 26.3 |

# References


1. Bevington P, Robinson DK. Data Reduction and Error Analysis for the Physical Sciences. McGraw-Hill Education; 2003.

2. Ljung L. System Identification: Theory for the User. Pearson Education; 1998.
